# Nonsecretory multiple myeloma, how to make a diagnosis?

**DOI:** 10.4103/0019-5413.58615

**Published:** 2010

**Authors:** Viroj Wiwanitkit

**Affiliations:** *Wiwanitkit House, Bangkhae, Bangkok Thailand 10160* E-mail: wviroj@yahoo.com

Editor,

I read the recent case report on nonsecretory multiple myeloma with a great interest.^1^ Middela et al. noted that nonsecretory multiple myeloma is a case with diagnostic dilemma and “a high index of suspicion must be borne in mind when excluding multiple myeloma as a cause of pain, pathological fracture or lytic lesion”.^1^ Indeed, it is accepted that diagnosis of nonsecretory multiple myeloma is very difficult. In this case, although there are some soft signs showing myeloma nature (decreased normal immunoglobulin and lytic bone lesion), the lack of abnormal secreted protein brings the diagnostic difficulty. Recently, the new laboratory technique for detecting of low level of abnormal secreted protein has been developed and can be the new hope in the fast and easy diagnosis of nonsecretory multiple myeloma. The “Freelite™ test” is an example. This test helps determine low level of abnormal free light chain and has been accepted as the new tool for diagnosis of non secretory multiple myeloma.^2^,^3^
